# Pls1 Is a Peroxisomal Matrix Protein with a Role in Regulating Lysine Biosynthesis

**DOI:** 10.3390/cells11091426

**Published:** 2022-04-22

**Authors:** Yotam David, Inês Gomes Castro, Eden Yifrach, Chen Bibi, Enas Katawi, Dekel Yahav Har-Shai, Sagie Brodsky, Naama Barkai, Tommer Ravid, Miriam Eisenstein, Shmuel Pietrokovski, Maya Schuldiner, Einat Zalckvar

**Affiliations:** 1 Department of Molecular Genetics, Weizmann Institute of Science, Rehovot 7610001, Israel; yotam.david@weizmann.ac.il (Y.D.); ines.castro@weizmann.ac.il (I.G.C.); eden.yifrach@weizmann.ac.il (E.Y.); bibichen2@gmail.com (C.B.); enasskat@gmail.com (E.K.); dekely@weizmann.ac.il (D.Y.H.-S.); sagie.brodsky@weizmann.ac.il (S.B.); naama.barkai@weizmann.ac.il (N.B.); miriam.eisenstein@weizmann.ac.il (M.E.); shmuel.pietrokovski@weizmann.ac.il (S.P.); 2 Department of Biological Chemistry, Hebrew University of Jerusalem, Jerusalem 91904, Israel; tommer.ravid@mail.huji.ac.il

**Keywords:** peroxisome, *Saccharomyces cerevisiae*, high-content screen, protein targeting, lysine, Lys1, saccharopine, Pex5

## Abstract

Peroxisomes host essential metabolic enzymes and are crucial for human health and survival. Although peroxisomes were first described over 60 years ago, their entire proteome has not yet been identified. As a basis for understanding the variety of peroxisomal functions, we used a high-throughput screen to discover peroxisomal proteins in yeast. To visualize low abundance proteins, we utilized a collection of strains containing a peroxisomal marker in which each protein is expressed from the constitutive and strong *TEF2* promoter. Using this approach, we uncovered 18 proteins that were not observed in peroxisomes before and could show their metabolic and targeting factor dependence for peroxisomal localization. We focus on one newly identified and uncharacterized matrix protein, Ynl097c-b, and show that it localizes to peroxisomes upon lysine deprivation and that its localization to peroxisomes depends on the lysine biosynthesis enzyme, Lys1. We demonstrate that Ynl097c-b affects the abundance of Lys1 and the lysine biosynthesis pathway. We have therefore renamed this protein Pls1 for Peroxisomal Lys1 Stabilizing 1. Our work uncovers an additional layer of regulation on the central lysine biosynthesis pathway. More generally it highlights how the discovery of peroxisomal proteins can expand our understanding of cellular metabolism.

## 1. Introduction

The role of peroxisomes in the break-down of fatty acids and reactive oxygen species is widely known [1]. Yet, it is little appreciated that these organelles play an essential role in the metabolism of various amino acids, lipids and other key metabolites [2,3]. Moreover, new discoveries of peroxisome functions demonstrate that this organelle also takes part in non-metabolic roles such as cellular stress responses [1]. As the list of peroxisomal functions steadily increases, it becomes crucial to identify the complete set of proteins making up the peroxisomal proteome (the peroxi-ome).

Although systematic efforts have been undertaken to define the complete yeast peroxi-ome [4,5], this task is not yet completed. One reason for many peroxisomal residents to remain elusive, is that peroxisomes dynamically alter their enzyme composition according to specific metabolic conditions [6], suggesting that sampling of peroxisomes in different environments would be necessary to grasp their entire composition. Additionally, peroxisomes are small, and many proteins that reside in them are of low abundance, making them difficult to detect. An example of one such protein is the fuzzy onions homolog 1 (Fzo1), that can only be visualized in peroxisomes when expressed under a strong promoter [7]. 

Hence, we set out to identify additional peroxisomal proteins of low abundance by screening a collection of yeast strains in which each yeast protein is fluorescently tagged and expressed using a constitutive, strong promoter. To capture condition-specific peroxisomal proteins we screened cells under two metabolic conditions. By using a high content visual approach, we identified 18 new peroxisomal proteins in yeast and systematically characterized their targeting pathways. Focusing on one of these proteins, Ynl097c-b, we found that this short peptide (40 amino acids) is generated from a gene that arose de novo in the *Saccharomyces cerevisiae* lineage. We show that Ynl097c-b is enriched in peroxisomes upon lysine deprivation and that its localization depends on the targeting factor Pex5 and the lysine biosynthesis enzyme, Lys1. Furthermore, we found that Ynl097c-b affects the abundance of Lys1 and lysine synthesis. Therefore, we named Ynl097c-b Pls1 for Peroxisomal Lys1 Stabilizing protein 1.

The identification of additional peroxisomal proteins sheds light on the number of potential peroxisomal functions and regulatory mechanisms that are yet to be characterized. Further exploration of them across different species could have a major impact on our understanding of the overall roles that peroxisomes hold in cells.

## 2. Materials and Methods

### 2.1. Yeast Strains and Strain Construction

*S. cerevisiae* strains were all based on the laboratory strain BY4741 [8] or a synthetic genetic array (SGA) compatible strain (YMS721) [9]. Primers for genetic manipulations and their validation were designed using Primers-4-Yeast (http://www.weizmann.ac.il/Primers-4-Yeast) [10] (Last accessed 25 November 2021). Polymerase chain reaction (PCR) products were transformed using the lithium-acetate method [11]. Following transformation, strains were verified by colony PCR for the correct insertion. All strains, plasmids, and primers used in this study are listed in Table S3, Table S4 and in Table S5, respectively.

### 2.2. Yeast Growth Media

Synthetic media used in this study contains 6.7 g/L yeast nitrogen base with ammonium sulfate (#1545 Conda Pronadisa, Madrid, Spain) and either 2% glucose (SD) or 0.2% oleic acid (Sigma-Aldrich, St. Louis, MO, USA) + 0.1% Tween 80 (Sigma-Aldrich, St. Louis, MO, USA) with complete amino acid mix (oMM composition [12]) unless written otherwise. When Hygromycin or Geneticin antibiotics were used, the media contained 0.17 g/L yeast nitrogen base without ammonium sulfate (#1553 Conda Pronadisa, Madrid, Spain) and 1 g/L of monosodium glutamic acid (Sigma-Aldrich, St. Louis, MO, USA #G1626). Yeast strains were selected using Hygromycin B (500 mg/L, Formedium, Swaffham, UK), Geneticin (G418) (500 mg/L, Formedium, Swaffham, UK), and Nourseothricin (200 mg/L, “ClonNat” WERNER BioAgents, Jena, Germany).

Lysine-deprived medium and amino acid depletion medium contained 1.7 g/L yeast nitrogen base without ammonium sulfate (#1553 Conda Pronadisa, Madrid, Spain) and 2% glucose with oMM composition [12], excluding only lysine or with a mix of 200 mg/L methionine, leucine, histidine, and uracil (Formedium, Swaffham, UK) correspondingly. 

SD-riboflavin medium was used to wash and image cells in high-throughput screens performed in glucose metabolic conditions to reduce autofluorescence. This medium contains 1.9 g/L yeast nitrogen base without ammonium sulfate, folic acid, and riboflavin (Formedium, Swaffham, UK), and complete amino acid mix (oMM composition, [12]), ammonium sulfate (Sigma-Aldrich, St. Louis, MO, USA) 5 g/L and 2% glucose.

### 2.3. Yeast Library Preparation

To create collections of haploid strains containing overexpressed (*TEF2* promoter) mCherry-tagged proteins with additional genomic modifications such as a peroxisomal marker (Pex3 tagged at the C-terminus with a Green Fluorescent Protein (GFP)) or different deletions (*Δpex5* and *Δpex7*), query strains were constructed based on an SGA compatible strain (for further information see Table S3). Using the SGA method [13,14], two collections were made. In the first, a query strain with the peroxisomal marker Pex3-GFP was crossed with a subset of the SWAT *TEF2*pr-mCherry library [15,16,17]. To create the second collection, three query strains–control, *Δpex5*, and *Δpex7*, were crossed with the hits from the first screen taken from the SWAT *TEF2*pr-mCherry library. SGAs were performed in a high-density format using a RoToR benchtop colony arrayer (Singer Instruments, Watchet, UK). In short, mating was performed on rich medium plates, and selection for diploid cells was performed on SD-URA plates containing query strain-specific antibiotics. Sporulation was induced by transferring cells to nitrogen starvation medium plates for 7 days. Haploid cells containing the desired mutations were selected by transferring cells to SD-URA plates containing the same antibiotics as for selecting diploid cells, alongside the toxic amino acid derivatives 50 mg/L Canavanine (Sigma-Aldrich, St. Louis, MO, USA) and 50 mg/L Thialysine (Sigma-Aldrich, St. Louis, MO, USA), to select against remaining diploids, and lacking Histidine to select for spores with an A mating type. 

### 2.4. Automated High-Throughput Fluorescence Microscopy

The resulting collections were imaged using an automated microscopy setup [18]. Briefly, query strains were transferred from 1536 format agar selection plates to liquid medium (SD-HIS + Nourseothricin) in 384-well polystyrene plates (Greiner, Kremsmünster, Austria) and grown overnight in a Liconic incubator at 30 °C. Strains were diluted to an OD_600_ of ~0.2 using a JANUS liquid handler (PerkinElmer, Waltham, MA, USA) in SD medium (6.7 g/L yeast nitrogen base and 2% glucose) or S-oleate (6.7 g/L yeast nitrogen base, 0.2% oleic acid, and 0.1% Tween-80) supplemented with complete amino acids. Plates were then incubated at 30 °C for 4 h in SD medium or for 20 h in S-oleate. After incubation, strains were transferred to 384-well glass-bottom microscope plates (Matrical Bioscience, Spokane, DC, USA) coated with Concanavalin A (Sigma-Aldrich, St. Louis, MO, USA) for 20 min. Wells were washed twice with SD-Riboflavin complete medium (for screens in glucose) or with double-distilled water (for screens in oleate) to remove non-adherent cells and obtain a cell monolayer.

Imaging of the initial screen for the identification of new peroxisomal proteins performed using a ScanR automated inverted fluorescent microscope system (Olympus) in SD-Riboflavin complete medium using a ×60 air lens (NA 0.9, GFP (490 nm), and RFP (572 nm)). 

Secondary screens were imaged using a Hamamatsu flash orca 4.0 camera and a CSU-W1 Confocal Scanner Unit of Yokogawa with a 50 µm pinhole disk. The software used was ScanR Olympus soft imaging solutions acquisition 3.2, and images were acquired using a 60× air lens (NA 0.9, GFP (488 nm), and RFP (561 nm)). 

All screens were imaged at 24 °C. For all micrographs, a single focal plane is shown. Image analysis was performed manually using ImageJ software [19].

### 2.5. Manual and High-Resolution Microscopy

Yeast strains were grown as described above for the high-throughput microscopy with changes in the selection required for each strain (See yeast strain information in Table S3). For imaging performed in amino acid depletion or lysine-deprived media, cells were washed twice with corresponding medium prior to imaging.

Imaging was performed at 30 °C using Olympus IXplore SpinSR system, composed of an Olympus IX83 inverted microscope scanning unit (SCU-W1) in addition to a high-resolution spinning disk module (Yokogawa CSU-W1 SoRa confocal scanner with double microlenses and 50-µm pinholes), operated by ScanR. Cells were imaged using a 60× oil lens (NA 1.42) and Hamamatsu ORCA-Flash 4.0 camera. Fluorophores were excited by a laser, and images were recorded in two channels: GFP (excitation wavelength 488 nm, emission filter 525/50 nm) and mCherry (excitation wavelength 561 nm, emission filter 617/73 nm). For all micrographs, a single focal plane is shown. 

For high-resolution imaging, cells were imaged using Z-stacks and deconvoluted in the cellSens software (Olympus, Shinjuku, Tokyo, Japan). Representative focal planes are shown. 

### 2.6. Image Analysis and Statistical Analysis

Image analysis was performed using ScanR analysis software (Olympus, Shinjuku, Tokyo, Japan). Thousands of cells derived from three independent biological repeats were segmented by artificial intelligence algorithms (ScanR Olympus soft imaging solutions, version 3.2). The cellular mean intensity (meaning the total cellular intensity divided by the area of the cell) of detected cells (GFP-Lys1 in Figure 6B and mCherry-Ynl097c-b in Figure S2A) or total intensity (used for small sub-objects where size is not accurately measurable, such as the case of mCherry-Ynl097c-b (Figure S2B)) were measured, and their mean was calculated for each experiment. Statistical analysis of the differences in GFP or mCherry intensity was performed using two-tailed t tests, assuming Gaussian distribution. Data analysis was performed using GraphPad Prism 9 (GraphPad Software Inc., San Diego, CA, USA).

### 2.7. Western Blotting and Quantitative Analysis

Cells were grown in glucose-containing media with appropriate selections overnight at 30 °C. Then, cells were diluted to 0.2 OD_600_ in SD medium with complete amino acid mix or lysine-deprived mix, and grown for an additional 4 h. At mid-log (0.5–0.8 OD_600_), a total of 5 OD_600_ cells were collected by centrifugation at 3000× *g* for 3 min, transferred to a fresh 1.5 mL microcentrifuge tube, and washed with 1 mL of nuclease-free water. Cells were resuspended in 200 µL lysis buffer (8 M urea, 50 mM Tris, pH 7.5, and protease inhibitors cocktail (Merck)) and subsequently lysed by vortexing at high speed with glass beads (Scientific Industries, Bohemia, New York) at 4 °C for 10 min. A total of 25 µL of 20% SDS was added to each sample before incubation at 45 °C for 15 min. The bottom of the microcentrifuge tubes was then pierced, loaded into 5 mL tubes, and centrifuged at 4000× *g* for 10 min to separate the lysate from the glass beads. The flow-through collected in the 5 mL tubes was transferred to a fresh 1.5 mL microcentrifuge tube and centrifuged at 20,000× *g* for 5 min. The supernatant was collected and 4× SDS-free sample buffer (0.25 M Tris, pH 6.8, 15% glycerol, and 16% Orange G containing 100 mM DTT) was added to the lysates, which were incubated at 45 °C for 15 min.

Protein samples were separated by SDS-PAGE using a 4–20% gradient gel (Bio-Rad, Hercules, California, CA, USA) and then transferred onto 0.45-µm nitrocellulose membrane (Pall Corporation, New York, NY, USA) using the Trans-Blot Turbo transfer system (Bio-Rad, Hercules, California, CA, USA). Membranes were blocked in SEA BLOCK buffer (Thermo Scientific, Waltham, MA, USA; diluted 1:5 in PBS) for 1 h at RT and subsequently incubated overnight at 4 °C with primary antibodies diluted in a 2% wt/vol BSA/PBS solution containing 0.01% NaN3. Primary antibodies used were rabbit anti-GFP (ab290, 1:3000; Abcam, Cambridge, UK), rabbit anti-Histone H3 (ab1791, 1:5000; Abcam, Cambridge, UK) and mouse anti-Hsp70 (ab5439 1:5000). After washing in TBST buffer, membranes were then probed with one or both of the following secondary antibodies: 800 CW Goat anti-Rabbit IgG (ab216773, 1:10,000, Abcam, Cambridge, UK) and IRDye 680 RD Goat anti-mouse (926-68070, 1:10,000, LI-COR, Lincoln, U.S.A) diluted in 5% wt/vol non-fat milk/Tris-buffered saline with 0.05% Tween 20 (TBST) for 1 h at RT. Blots were washed and imaged on the Odyssey Infrared Scanner (LI-COR, Lincoln, NA, USA). Quantitative analysis of the blots was performed by detecting band intensity using GelAnalyzer 19.1 (www.gelanalyzer.com) (Last accessed 9 November 2021). Values of detected bands representing GFP were normalized to the Histone loading control. Statistical analysis of the differences was done using two-tailed t tests, assuming Gaussian distribution. Data analysis was performed using GraphPad Prism 9 (GraphPad Software Inc., San Diego, CA, USA).

### 2.8. Whole Cell Metabolomics

Yeast strains were grown overnight in minimal synthetic medium, and triplicates were diluted to 0.5 OD_600_ in breathable plastic tubes (LIFEGENE) containing lysine-deplete medium (recipe detailed above). After 4 h, OD_600_ values were measured, and cells were harvested. Each sample was filtered using GVS Magna™ Nylon Membrane Filters. The sample-containing filter was washed using double distilled water and transferred into a preservation mix (50:50) double distilled water: HPLC-grade acetonitrile (Sigma-Aldrich, St. Louis, MO, USA) placed at −80 °C until measurement. Sample analysis was carried out by MS-Omics as follows. The analysis was carried out using a Thermo Scientific Vanquish LC coupled to Thermo Q Exactive HF MS. An electrospray ionization interface was used as an ionization source. Analysis was performed in negative and positive ionization modes. The UPLC was performed using a slightly modified version of the following protocol [20]. Peak-integration was performed as a two-legged approach: an automatic approach using Compound Discoverer 3.1 (ThermoFisher Scientific, Waltham, MA, USA) and a manual peak integration using Skyline [21]. Data from the two approaches were subsequently merged into one dataset, with compounds detected by Compound discoverer having compound id starting with X, and compounds from skyline starting with SL. Identification of compounds was performed at four levels; Level 1 (based on the following parameters matched against an in-house library): retention times, accurate mass (with an accepted deviation of 3 ppm), and MS/MS spectra, Level 2a (based on the following parameters matched against an in-house library): retention times, and accurate mass (with an accepted deviation of 3 ppm). Level 2b (based on the following parameters matched against an external library): identification by accurate mass (with an accepted deviation of 3 ppm), and MS/MS spectra. Statistics were performed as unpaired t-test with Benjamini-Hochberg corrections (with accepted false discovery rate at 5%) and calculations of log2 ratios were performed. All calculations were performed in Excel.

### 2.9. Binding Predictions Using ANCHORSmap

The structure of Lys1 in its active closed state, bound to NADH and saccharopine (PDB entry 3UH1), was used to detect putative lysine binding locations using ANCHORSmap (Ben-Shimon and Eisenstein, 2010). ANCHORSmap searches for anchoring spots, namely depressions on the surface of one protein with bound single amino acid of another protein. The binding energy (ΔG) is calculated taking into consideration that the bound amino acid is part of a protein. 

### 2.10. Computational Phylogenomics

The presence of the *YNL097C-B*, (*PLS1*) gene in *Sacchoromyces cerevisiae* was detected by blast [22] searches in the public NCBI genome databases, using its product from the S288C strain as query. Such searches also showed the absence of the gene in all other species with publicly available genomes data. To further verify this in other yeast species, we used the coding region of the gene and its 5- and 3-untranslated region (extended all the way to the *PHO23* and *RAS2* genes that flank Pls1) in *Sacchoromyces cerevisiae* strains as query for blast searches in yeast where these two flanking genes are syntenous to *Sacchoromyces cerevisiae*, i.e., are present in the same orientation and relative positions to each other. The phylogenetic tree was calculated by the phyml program [23] using default parameters and 100 bootstrap analysis repeats, and the trees were drawn using FigTree (http://tree.bio.ed.ac.uk/software/figtree/) accessed on 25 November 2018). Sequence logos [24] were calculated from multiple alignments as previously described [25].

## 3. Results

### 3.1. A High Content Screen Reveals Condition-Specific Peroxisomal Proteins

**Figure 1:**
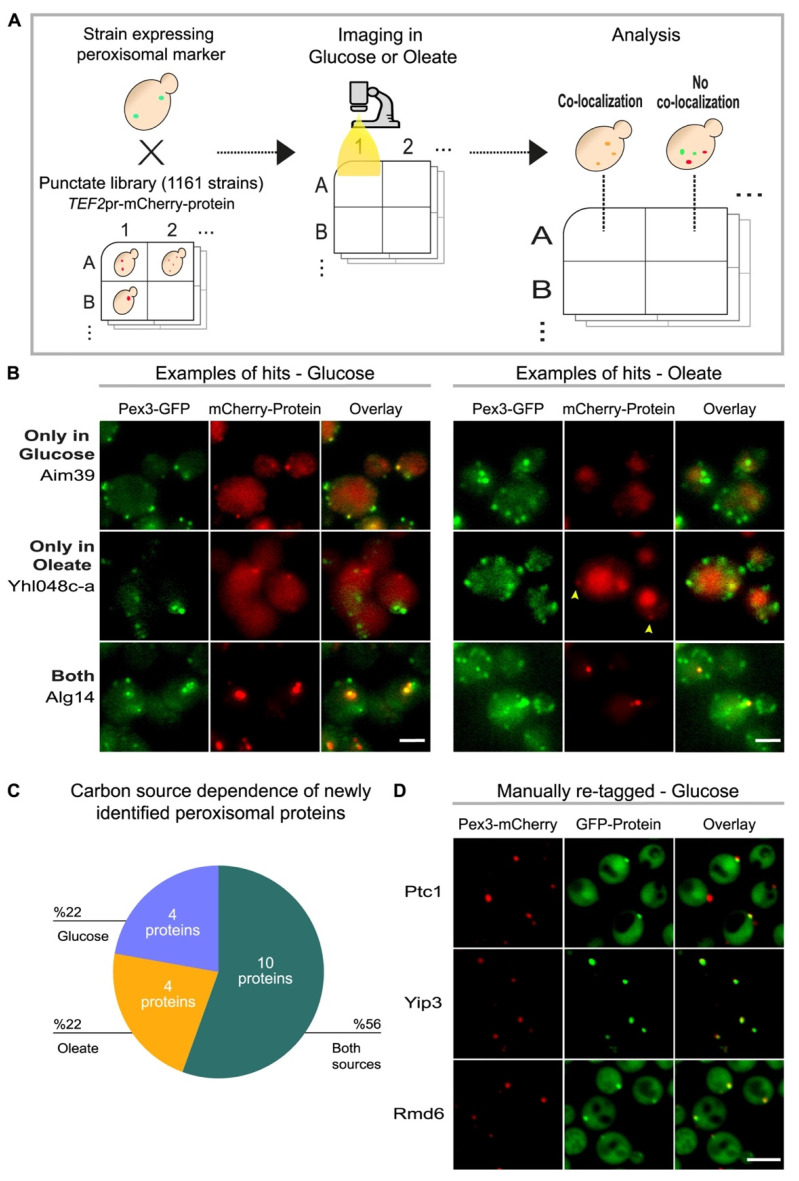
A high-throughput screen uncovers peroxisomal residents dependent on metabolic status. (**A**) Schematic illustration of the high-content screen performed to discover low-abundance peroxisomal proteins. A strain (mating type A) with peroxisomal marker (Pex3-GFP) was crossed with a subset of the *TEF2*pr-mCherry library (mating type alpha), made of 1161 yeast strains in which each strain has a single gene expressed from the strong and constitutive *TEF2*-promoter and N-terminally tagged with mCherry. The localization of all proteins in this subset had been previously annotated as puncta. Sporulation was induced and haploids containing both traits (the peroxisomal marker and a single protein tagged with mCherry) were selected. Automated imaging of the arrayed collection was performed in medium with either glucose or oleate as carbon sources, and the resulting images were analyzed manually for tagged proteins that co-localized with the peroxisomal marker. (**B**) Fluorescent images of three examples of proteins that came up in the screen as co-localized with the peroxisomal marker (Pex3-GFP). mCherry-Aim39 is an example of a protein that only co-localized with peroxisomes in glucose and mCherry-Yhl048c-a of those that only co-localized in oleate. mCherry-Alg14 is an example of a protein that co-localized with peroxisomes in both conditions. Scale bar, 5 µm. (**C**) Pie chart summarizing the newly identified candidate peroxisomal proteins in each condition. (**D**) Fluorescent images of selected proteins that were further validated by remaking the tagged strains. Ptc1, Yip3 and Rmd6 were expressed from the *TEF2*-promoter and tagged with GFP. Peroxisomes were visualized by Pex3 tagged with mCherry. All three manually retagged proteins maintained their co-localization with Pex3. Full information on the proteins that came up in the screen and which proteins were validated can be found in Table S2. Scale bar, 5 µm.

To discover low abundance peroxisomal proteins, we used a collection of *Saccharomyces cerevisiae* (hereafter called *S. cerevisiae* or yeast) strains (library) in which each native promoter was genomically replaced by a strong and constitutive *TEF2* promoter followed by the sequence of the mCherry protein in frame with the gene [16,17]. To minimize the size of the experimental setup and maximize the chances of finding peroxisomal proteins, we collected 1161 strains in which the protein localization was annotated as puncta, a similar pattern to how peroxisomes are visualized in regular fluorescence microscopes [15] (Table S1). We integrated a peroxisomal marker (Pex3-GFP) into this collection to differentiate between peroxisomes and other punctate structures. Since some proteins are localized to peroxisomes only under specific conditions [1], we imaged these strains in two different carbon sources: glucose-containing medium, the standard growth condition for yeast, and in oleate as a sole carbon source, where peroxisomes are essential, more active, and abundant. We then analyzed each strain to identify proteins that co-localize with the peroxisomal marker (Figure 1A).

Our screen revealed a total of 18 candidate proteins that co-localized with peroxisomes in either carbon source (Table S2). While ten proteins localized to peroxisomes in both metabolic conditions, four only localized to peroxisomes in glucose, and an additional four proteins only when cells were grown in oleate (Figure 1B). The observation that the peroxisomal localization of certain proteins depends on the metabolic state of the cell (Figure 1C) is supported by our previous observations [4]. Since all candidate proteins were constitutively expressed, it is plausible that the colocalization with peroxisomes in different metabolic conditions was regulated post-translationally.

Since high content imaging of entire libraries can give rise to both false positives and false negatives, our 18 proteins must be validated before they can be classified as bona fide peroxisomal proteins. To verify a subset of the newly identified proteins, we manually retagged several of the strains and confirmed the identity of the tagged proteins (in total eight of the 18 proteins were validated either by retagging and PCR or only by PCR, see Table S2 for verification information on each strain). For three proteins, Ptc1, Yip3 and Rmd6, we retagged the proteins at the N-terminus with GFP and expressed them under the same *TEF2* promoter in strains where peroxisomes were marked with Pex3-mCherry to ensure that the specific tag was not influencing protein localization. Indeed, we could verify their peroxisomal localization (Figure 1D).

One of the newly identified proteins is Ptc1 (protein serine/threonine phosphatase 1), a type 2C phosphatase with a predicted mitochondrial targeting sequence [26] that was shown to control the inheritance of peroxisomes and mitochondria [27,28]. Since Ptc1 has a potential peroxisomal targeting signal type 1 (PTS1) at its most C-terminus [29], it has a strong probability of being a bona fide peroxisomal protein. Interestingly, Ptc5, another type 2C phosphatase, was recently shown to have dual targeting to mitochondria and peroxisomes [30], suggesting a conserved mechanism for dual targeting to peroxisomes and mitochondria for some of the type 2C phosphatase family members.

Unexpectedly, we identified the Golgi and endosomal protein Yip3 (Ypt-interacting protein 3) at peroxisomes. Yip3 is known to promote the dissociation of endosomal Rab–guanine nucleotide dissociation inhibitor (GDI) complexes [31]. It was therefore unclear why it would colocalize with peroxisomes. 

Last, we validated Rmd6 (required for meiotic nuclear division 6), a protein whose biological role and cellular location were unknown. Sequence analyses of the C-terminus of Rmd6 suggest it may possess a PTS1 motif (Histidine, Arginine, Leucine: HRL). This particular sequence has been previously predicted and shown to be a functional targeting sequence [29,32]. The exact function of Rmd6 in peroxisomes remains to be discovered. 

Altogether, our results further expand the knowledge on the complete peroxi-ome and demonstrate that the metabolic state of the cell alters it.

### 3.2. A Systematic Approach Uncovers Targeting Factor Dependency of the Newly Identified Peroxisomal Proteins

**Figure 2:**
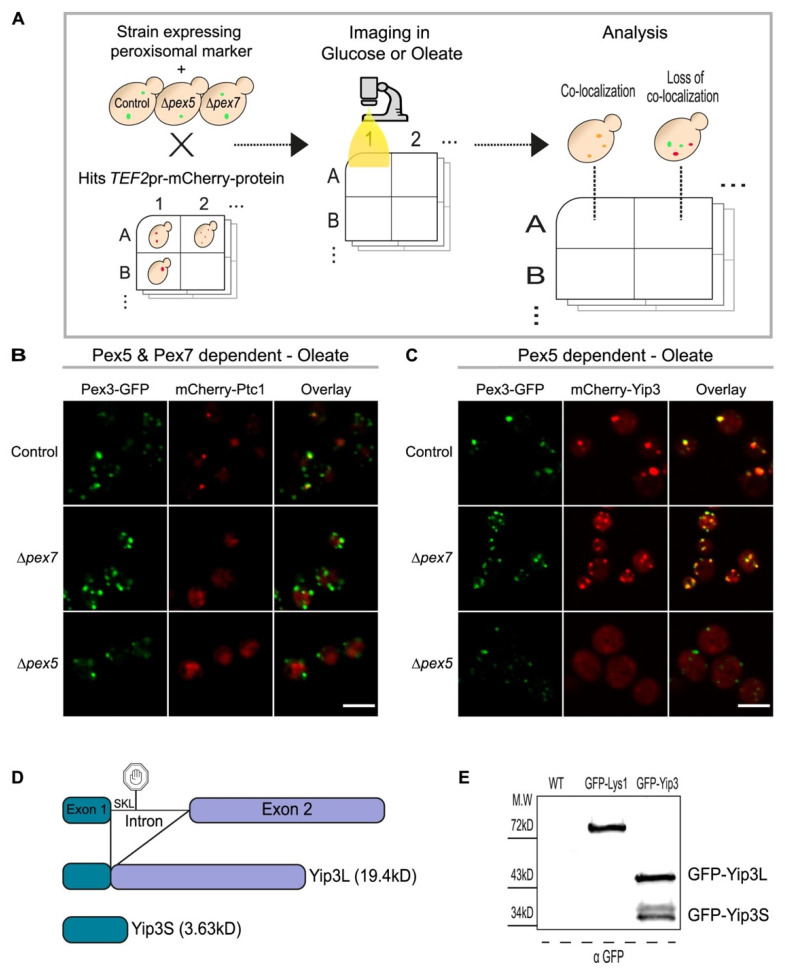
A systematic approach uncovers targeting factor dependency for the newly identified peroxisomal proteins. (**A**) Schematic illustration of the high content screen performed to discover the targeting factor dependency of the newly identified peroxisomal proteins. Automated mating procedures were used to integrate a strain (mating type A) with either only a peroxisomal marker (Pex3-GFP) or with additional deletion of either *PEX5* or *PEX7* into a mini-library (mating type alpha) made from all newly identified peroxisomal candidates. Sporulation was induced and haploids containing all desired traits (the peroxisomal marker and a single protein tagged with mCherry for control strains or with additional deletion of *PEX5* or *PEX7*) were selected. Automated imaging was performed in medium with either glucose or oleate as a carbon source. The resulting images were analyzed manually for deletions affecting protein signal and localization. (**B**) Fluorescent images showing an example of a protein, Ptc1, that is dependent on both Pex5 and Pex7 for peroxisomal targeting in oleate. Both deletions of *PEX5* or *PEX7* affect the peroxisomal localization of mCherry-Ptc1. Scale bar, 5 µm. (**C**) Fluorescent images showing an example of a protein that is dependent on Pex5 for peroxisomal targeting in oleate. In both control and *∆pex7* strains mCherry-Yip3 co-localizes with the peroxisomal marker (Pex3-GFP). However, in the absence of Pex5, mCherry-Yip3 is localized to the cytosol suggesting that Yip3 is a Pex5 cargo. Scale bar, 5 µm. (**D**) A schematic illustration of the *YIP3* locus. A splicing event gives rise to a short Yip3 variant (3.6 kD) which harbors a PTS1 sequence at the C-terminus. (**E**) Western blot analysis demonstrates the shorter variant of Yip3. Proteins from wild type, GFP-Lys1 (control), and GFP-Yip3 strains were extracted and subjected to SDS-PAGE and immunoblotting against GFP. Two bands of GFP-Yip3 were observed corresponding to the molecular weight calculated for the long variant 45.4 kD (Yip3L) and the short variant 29.6 kD (Yip3S) (with the addition of a 26 kDa GFP fluorophore).

Of the 18 newly identified candidate peroxisomal proteins, eight were predicted to be membrane-spanning, and ten were predicted as soluble proteins [33]. Two main targeting factors transport soluble proteins to the peroxisome matrix: Pex5 primarily targets proteins containing a PTS1 motif [34,35], and Pex7 targets proteins containing a PTS2 [36]. From the ten soluble proteins, three were previously predicted to possess a PTS1 motif [29] (See Table S2) while the remaining seven have no identified PTS1 or PTS2 motifs. To systematically identify the cargo factor on which each newly identified peroxisomal protein relies, we screened these proteins on the background of either a control strain containing only a peroxisomal marker (Pex3-GFP) or with an additional *PEX5* or *PEX7* deletion (Figure 2A). 

We and others have previously shown that it is possible to microscopically determine the cargo dependency by loss of peroxisomal signal on the background of a deletion in the targeting factor [4,5,30,37].

We found several proteins that were independent of Pex5 or Pex7 (the complete list is in Table S2), all were predicted to contain transmembrane domains as expected. Deletion of *PEX7* affected the localization of only one protein, Ptc1, and solely in oleate growth conditions. In contrast, the deletion of *PEX5* influenced Ptc1 targeting in both growth conditions (Figure 2B). While in mammals, Pex7 requires Pex5 for protein targeting, the two targeting factors are assumed to be independent in yeast [6], therefore this result was unexpected. A possible explanation is that by tagging Ptc1 at the N-terminus, we inadvertently altered the properties of its mitochondrial targeting sequence [26] to resemble a PTS2-like sequence and enabled targeting by Pex7. The fact that this only occurred in oleate may suggest that this requires the oleate-specific Pex7 adapter, Pex21 [38]. Regardless it is not likely that Ptc1 is a native cargo of Pex7.

Of the newly identified proteins, 11 were dependent on the presence of Pex5 either in one or both conditions. Since most of these proteins do not contain known PTS1 motifs, their targeting may be mediated by alternative binding methods such as a non-canonical PTS1 or by binding to other regions in Pex5 that differ from the PTS1 binding groove [39].

Interestingly, some of these proteins were predicted to have transmembrane domains. Although unexpected, this has been previously shown for Pex14 in yeast [40] and for ATGL (adipose triglyceride lipase) in mammalian cells [41]. From these we chose to examine the case of Yip3. Being a well-studied protein that is known to span the membrane [31,42] its clear dependence on Pex5 (Figure 2C) could be a phenomenon similar to Pex14. However, we noticed that the DNA sequence of *YIP3* possesses an intron, an infrequent event in the yeast genome [43]. Sequence analysis showed that if the intron would not be spliced out, the transcript would encode an alternative protein whose last three amino acids (Serine, Lysine, Leucine: SKL) are a canonical PTS1 [6] (Figure 2D). In yeast, unlike in mammals [44] and filamentous fungi [45], a shorter Yip3 protein has never been described. To uncover whether Yip3 has a short peroxisomal matrix isoform that would explain the requirement for Pex5 targeting, we analyzed the protein product of an N-terminally tagged GFP-Yip3 by immunoblotting. Indeed, we found that two isoforms exist in nearly equal levels—a full-length form (19.4 kD) that we now call Yip3 Long (Yip3L) and a shorter isoform (3.6 kD) that we now call Yip3 Short (Yip3S) (Figure 2D,E). The role of Yip3S in the peroxisome matrix now remains to be investigated.

### 3.3. Ynl097c-b Is a Newly Identified Peroxisomal Protein That Is Unique to Saccharomyces cerevisiae

**Figure 3:**
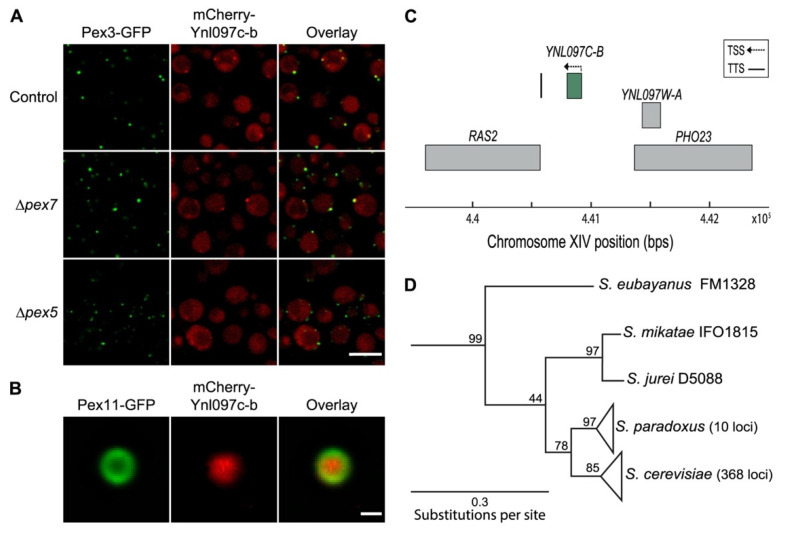
Ynl097c-b is a peroxisomal protein unique to *S. cerevisiae.* (**A**) Fluorescent images showing mCherry-Ynl097c-b co-localizing with Pex3-GFP in control cells and in the absence of *PEX7*, but not in the absence of *PEX5*, implying that it is a Pex5 cargo. Scale bar, 5 µm. (**B**) High-resolution images of *TEF2*pr-mCherry-Ynl097c-b. A strain with enlarged peroxisomes was created by tagging Pex11 at the C-terminus with GFP, and cells were grown in amino acid depletion medium. The signal of mCherry-Ynl097c-b fills the matrix of peroxisomes which is surrounded by a ring-like membrane signal of Pex11-GFP. Scale bar, 500 nm. (**C**) Schematic representation of the genomic area harboring the *YNL097C-B* gene between *PHO23* and *RAS2*. The transcription start site (TSS) is marked by an arrow and the transcription termination site (TTS) by a solid line (site coordinates taken from [46]). (**D**) Phylogenetic tree of the *PHO23* and *RAS2* coding region loci from various yeast species. The *S. cerevisiae* and *S. paradoxus* loci are each collapsed. *S. paradoxus* is the closest species to *S. cerevisiae*, with a high confidence level (bootstrap value of 78/100). The scale bar shows substitutions per site, bootstrap values (out of 100 repeats) are shown for the junctions.

From our 18 candidate peroxisomal proteins, we chose to focus on one of the newly identified proteins, Ynl097c-b, which is a 40 amino acid-long peptide with an uncharacterized function. We found that the peroxisomal localization of Ynl097c-b in glucose medium is dependent on Pex5 (Figure 3A). Since this is a soluble proteins it suggests that it localizes to the peroxisome matrix. 

To verify that Ynl097c-b resides in the matrix of peroxisomes, we used an imaging-based approach developed in our lab that enables the detection of the sub-organellar distribution of peroxisomal proteins [5]. In this approach, peroxisomes are enlarged by growth in specific metabolic conditions (oleate or amino acid depletion), coupled with deletion or C-terminally tagging of Pex11 and imaging by high-resolution microscopy. Using this approach, we found that fluorescently tagged Ynl097c-b is indeed localized to the peroxisome matrix (Figure 3B). 

Despite being short, Ynl097c-b is clearly a protein-encoding gene as it has well-defined transcription start and termination sites (TSS and TTS) [46] (Figure 3C). To uncover potential functions for Ynl097c-b, we searched for homologs in other organisms. However, searches in extensive sequence databases found Ynl097c-b to appear exclusively in *S. cerevisiae*. This indicates *YNL097C-B* as a de novo appearing gene in baker’s yeast. *YNL097C-B* resides in a region between two well-characterized genes, *PHO23* (phosphate metabolism protein 23) and *RAS2* (ras-like protein 2) on chromosome 14 (Figure 3C). These two genes are also adjacent in other budding yeasts. Examining this intergenic region in all available yeast sequences confirmed the presence of the *YNL097C-B* gene only in *S. cerevisiae* and its hybrids. Examination of 857 *S. cerevisiae* loci revealed that the Ynl097c-b protein is highly conserved, with 89% of the proteins being identical or with one amino acid change to the protein in our lab strain (S288c) (Figure S1A). Using the *PHO23* and *RAS2* coding regions, we found that in this locus, the closest species to *S. cerevisiae* is *S. paradoxus* (Figure 3D) [47]. However, even *S. paradoxus* does not possess the *YNL097C-B* sequence in the corresponding region. In *S. cerevisiae* this region has two insertions of one and two bases, and 5′ initiator Methionine (ATG) and 3′ stop codons (Figure S1B).

Altogether our data suggest that Ynl097c-b is a Pex5-dependent peroxisomal matrix protein that appeared in the intergenic region between *RAS2* and *PHO23* genes in the *S. cerevisiae* species.

### 3.4. The Peroxisomal Localization of Ynl097c-b Is Dependent on Lysine Levels

**Figure 4:**
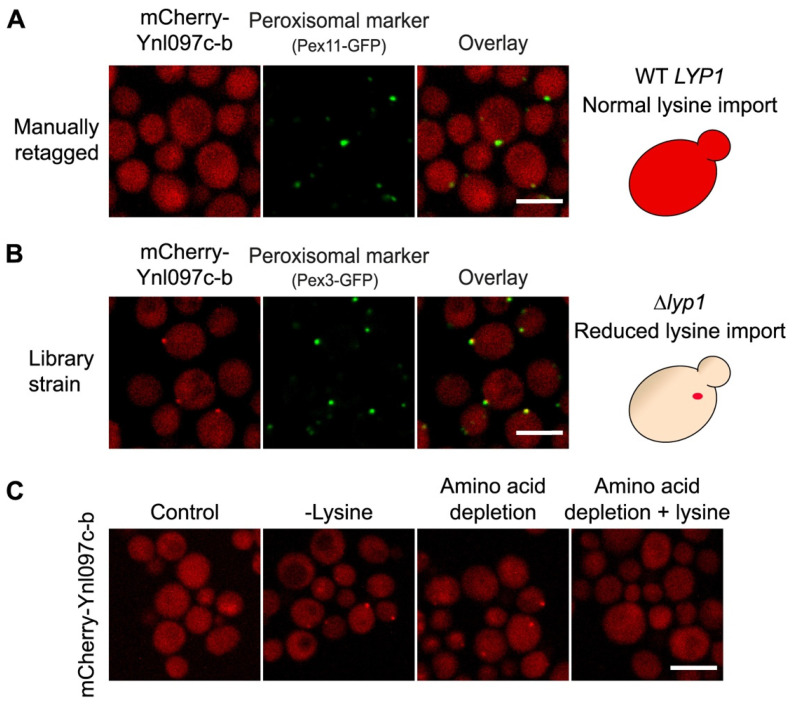
Ynl097c-b localization to peroxisomes is dependent on lysine levels. (**A**) Fluorescent images of a BY4741-based strain, with a WT *LYP1* gene, expressing manually tagged mCherry-Ynl097c-b and a peroxisomal marker (Pex11-GFP) imaged in glucose-containing medium. The signal of mCherry-Ynl097c-b (red in the right-hand scheme) is cytosolic. Scale bar, 5 µm. (**B**) Fluorescent images of the library strain with a *LYP1* deletion and expressing mCherry-Ynl097c-b. In this strain, colocalization with the peroxisomal marker (Pex3-GFP) is seen when imaged in glucose-containing medium. Scale bar, 5 µm. (**C**) Fluorescent images of a strain expressing mCherry-Ynl097c-b in different media; Control (glucose, complete amino acids), -Lysine (medium without lysine), amino acid depletion, and amino acid depletion with only lysine added. The punctate accumulation of mCherry-Ynl097c-b was observed in both conditions when lysine was absent. Scale bar, 5 µm.

Since our original strain background contains several metabolic markers that enable high-throughput manipulations, it was important to manually retag and follow up on Ynl097c-b localization in a standard laboratory strain (BY4741), devoid of these markers. Surprisingly, when we imaged the retagged strain, Ynl097c-b did not localize to peroxisomes, despite the use of the same fluorophore, promoter, and medium as in the screen (Figure 4A).

Since the only difference between experiments was the genetic background of the strains, we looked at the genes that were altered between these strains. While the retagged BY4741-based strain contained the *LYP1* (lysine-specific permease 1) gene (the only lysine permease in *S. cerevisiae*), it was deleted in the original screening strain, making it flawed in its ability to import lysine from the medium. This led us to hypothesize that the levels of cellular lysine could regulate the localization of Ynl097c-b to peroxisomes. 

To test our hypothesis, we exposed the retagged strain to media with or without lysine (Figure 4C). While in the standard medium that contains lysine, Ynl097c-b was localized to the cytosol, lysine depletion resulted in a punctate signal of the protein. To specifically establish the role of lysine as the cause for Ynl097c-b localization to peroxisomes, cells were incubated in amino acid deprivation medium. In these conditions, the signal of Ynl097c-b was punctate, while the addition of lysine alone was sufficient to relocalize the protein to the cytosol (Figure 4C). 

Our results demonstrate that lysine depletion is both necessary and sufficient to cause peroxisomal targeting of Ynl097c-b.

### 3.5. The Peroxisomal Localization of Ynl097c-b Depends on Lys1

Our observation that Ynl097c-b localizes to the peroxisomal matrix in a Pex5-dependent manner but lacks a PTS1 intrigued us to understand how it is targeted. One way by which peroxisome matrix proteins lacking targeting signals can be imported to peroxisomes is via a “piggybacking” mechanism, where they interact with another peroxisomal protein that directly binds the targeting factor [48,49]. In *S. cerevisiae*, the last step of lysine biosynthesis occurs in peroxisomes by the saccharopine dehydrogenase enzyme, Lys1 (Lysine requiring 1) [50], which possesses a PTS1 motif with high affinity to Pex5 [51]. Hence, our observation that Ynl097c-b localizes to peroxisomes when cells are cultured in the absence of lysine, a condition that is likely to increase Lys1 activity, led us to hypothesize that Ynl097c-b may rely on Lys1 for peroxisomal targeting.

**Figure 5:**
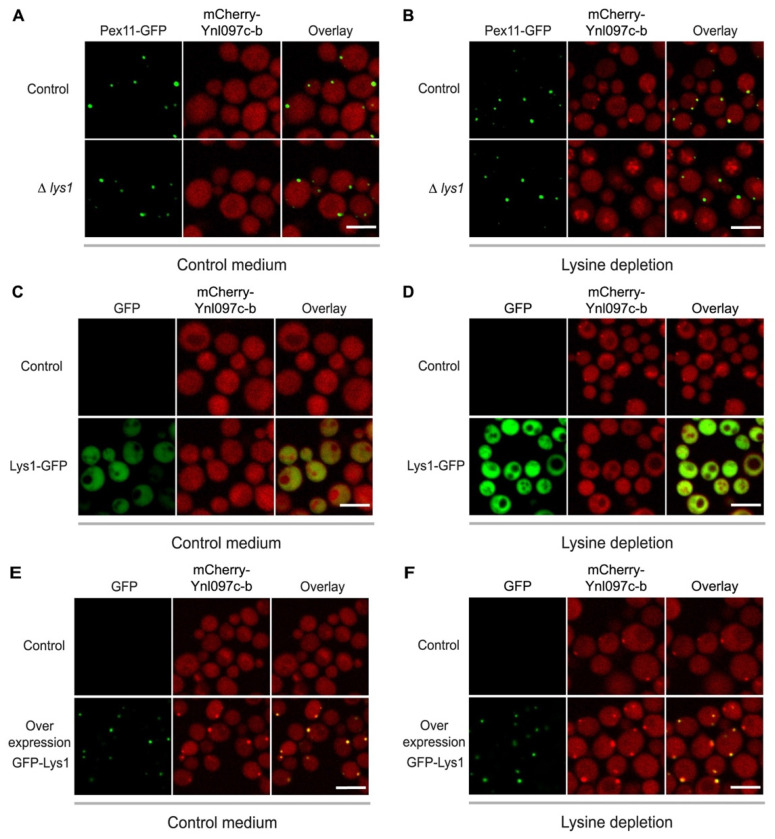
The targeting of Ynl097c-b to peroxisomes depends on Lys1. (**A**) Fluorescent images of a control strain with mCherry-Ynl097c-b and a peroxisomal marker (Pex11-GFP) compared to a strain with additional deletion of *LYS1* in control medium. When lysine is present in the media, Ynl097c-b remains cytosolic in both strains. (**B**) Fluorescent images of the same strains as in A, only grown in medium without lysine. mCherry-Ynl097c-b does not co-localize with the peroxisomal marker in the absence of Lys1, suggesting that this protein is required for the peroxisomal localization of mCherry-Ynl097c-b. Scale bar, 5 µm. (**C**) Fluorescent images of a control strain with mCherry-Ynl097c-b compared to a strain where Lys1 was additionally tagged at the C-terminus (masking its PTS1) in control medium. Ynl097c-b remains cytosolic regardless of C-terminus tagging in Lys1. Scale bar, 5 µm. (**D**) Fluorescent images of the same strains as in C, taken when cells were grown in lysine-depleted medium. In contrast to the control strain, following the mistargeting of Lys1 to the cytosol, Ynl097c-b does not co-localize with peroxisomes, implying that the peroxisomal localization of Ynl097c-b relies on the ability of Lys1 to be targeted to peroxisomes. Scale bar, 5 µm. (**E**) Fluorescent images of a control strain with only mCherry-Ynl097c-b compared to a strain in which Lys1 is expressed under the strong *TEF2* promoter and tagged at the N-terminus with GFP. In lysine-containing medium, mCherry-Ynl097c-b was localized to puncta only when Lys1 was overexpressed. Scale bar, 5 µm. (**F**) Fluorescent images of the same strains as in E, but imaged in medium lacking lysine. In these conditions, while overall Ynl097c-b signal does not increase (quantified in Figure S2A), the peroxisomal signal of Ynl097c-b becomes more robust when Lys1 was overexpressed (quantified in Figure S2B). This further strengthens the dependency of the peroxisomal localization of Ynl097c-b on Lys1. Scale bar, 5 µm.

To test this hypothesis, we first examined the localization of Ynl097c-b in the absence of Lys1. Indeed, in *LYS1*-deleted cells, Ynl097c-b did not localize to peroxisomes in neither control nor lysine-deprived media (Figure 5A,B). Moreover, in lysine deprivation the deletion of *LYS1* altered the signal of mCherry-Ynl097c-b to a vacuolar-like signal, which suggests the vacuolar degradation of mCherry-Ynl097c-b (Figure 5A). 

To examine if Ynl097c-b relies on the presence of Lys1 or on its targeting to peroxisomes, we masked the PTS1 motif of Lys1 by fusing a GFP to its C-terminus. This has been previously shown to prevent Pex5-recognition and result in Lys1 localization to the cytosol [50]. In this condition, both Lys1 and Ynl097c-b were localized to the cytosol (Figure 5C,D). Since the complete loss of Lys1 leads to lysine starvation, yet cytosolic localization (caused by masking the PTS1) of Lys1 enables lysine production [50], these results suggest that the peroxisomal localization of Ynl097c-b is a direct effect of Lys1 targeting to peroxisomes, rather than an indirect effect of lysine depletion. Indeed, when we overexpressed Lys1, Ynl097c-b was localized to peroxisomes regardless of the media (Figure 5E,F). Moreover, even though Ynl097c-b is constitutively expressed in both strains, in medium lacking lysine, the peroxisomal signal of mCherry-Ynl097c-b was stronger compared to a strain with endogenously-expressed Lys1 (Figure 5F and Figure S2B) although its overall abundance was not altered (Figure S2A). Therefore, it seems that elevated Lys1 levels cause increased targeting of Ynl097c-b to peroxisomes.

Altogether, our results demonstrate that the targeting of Ynl097c-b to peroxisomes depends on the presence of Lys1 and on its ability to be adequately targeted to peroxisomes.

### 3.6. Ynl097c-b Affects Lys1 Abundance and Lysine Biosynthesis

**Figure 6:**
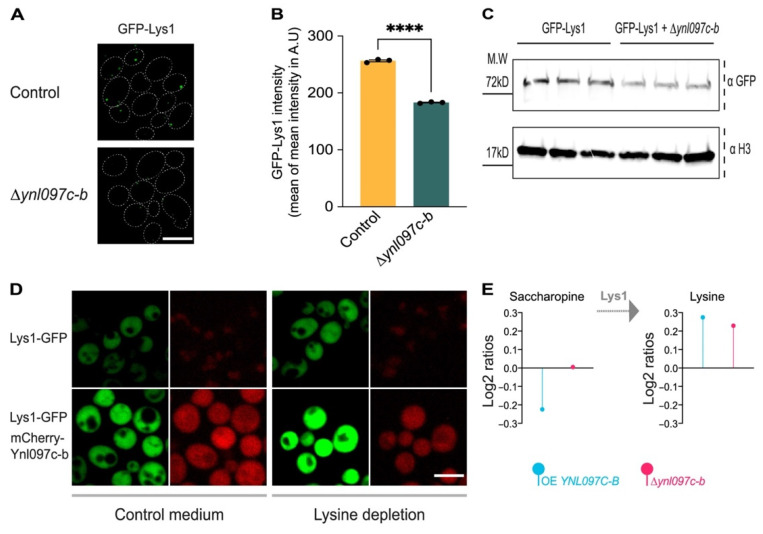
Ynl097c-b affects Lys1 abundance and lysine synthesis. (**A**) Fluorescent images of GFP-Lys1 in a control strain and in deletion of *YNL097C-B* in the absence of lysine. The absence of Ynl097c-b causes a reduction in the signal intensity of GFP-Lys1. Scale bar, 5 µm. Dotted lines represent individual cells. (**B**) Quantification of strains used in (**A**) for GFP-Lys1 levels (calculated as cellular mean intensity in arbitrary units (A.U)) in the presence or absence of Ynl097c-b. Data are presented as the mean calculated for each of three experimental repeats, each containing hundreds of individual cells. Bars represent mean with standard error from the three experimental repeats, **** *p* < 0.0001. (**C**) Western blot analysis of the same strains shown in (**A**). Three biological repeats were sampled, and immunoblotting was performed against GFP and Histone H3 as a loading control. The reduction in Lys1 levels in the absence of Ynl097c-b further supports the imaging-based experiments. For quantitative analysis of the blot see Figure S3. (**D**) Fluorescent images of Lys1-GFP in the control cells or in the presence of overexpressed mCherry-Ynlc097c-b, in medium with and without lysine. In both media, the overexpression of Ynl097c-b increased the signal intensity of Lys1-GFP. This effect was further highlighted in lysine-deprived medium. This observation supports the idea that Lys1 is stabilized by Ynl097c-b. Scale bar, 5 µm. (**E**) Bar graph of saccharopine (the immediate precursor to lysine) and lysine levels measured in overexpression (blue) or deletion (pink) strains of *YNL097C-B*. Three independent biological repeats of whole-cell extracts were sampled from cells exposed to lysine-deprived medium and analyzed by mass-spectrometry. The decrease in saccharopine and increase in lysine when *YNL097C-B* is overexpressed suggest that Ynl097c-b promotes lysine synthesis. The deletion of *YNL097C-B* only increased lysine levels, suggesting that other mechanisms may act to maintain cellular lysine in the measured conditions.

To further investigate the role of Ynl097c-b in lysine metabolism and its relationship to Lys1, we examined whether modulating the expression of Ynl097c-b affected Lys1 abundance. We found that in the absence of Ynl097c-b, the cellular mean intensity of GFP-Lys1 was significantly reduced compared to a control strain (Figure 6A,B).

Western blot analysis of GFP-Lys1 protein levels confirmed its decrease in the absence of Ynl097c-b (Figure 6C and quantified in Figure S3). In addition, overexpression of Ynl097c-b increased the signal intensity of C-terminally tagged Lys1, an effect that was stronger in lysine-deprived medium (Figure 6D).

The increased signal of Ynl097c-b in peroxisomes in lysine-deprived medium, its dependence on Lys1 for targeting, and its effect on Lys1 levels led us to hypothesize that Ynl097c-b may play a role in the regulation of lysine metabolism. To examine this, we performed metabolomic analysis of whole cells when *YNL097C-B* was overexpressed or deleted (see Table S6 for all metabolomic results). In support of the above, we saw that the overexpression of Ynl097c-b reduced the levels of saccharopine, the direct substrate of Lys1 [52], while lysine levels increased compared to the control (Figure 6E), suggesting enhanced levels or activity of Lys1. The deletion of Ynl097c-b only increased lysine levels, this might be due to a compensatory mechanism as a response to lower levels of Lys1 following Ynl097c-b deletion.

## 4. Discussion

Peroxisomes are metabolically active organelles that mediate various functions depending on the species and environmental conditions [1]. Therefore, it is not surprising that great efforts have been undertaken to characterize their complete proteome and function [53]. Our work complements these efforts by identifying proteins of low abundance or whose localization is affected by the metabolic state of the cell. 

**Figure 7:**
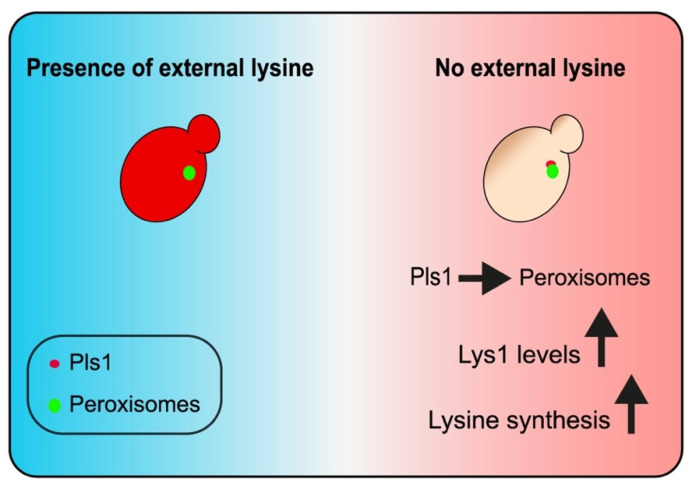
A schematic figure summarizing the key findings of this study. In the presence of external lysine, the signal of Pls1 (red) localizes mainly to the cytosol. However, in lysine-deprived conditions, Pls1 is localized to peroxisomes (green) depending on the presence of Lys1. In these conditions, Pls1 contributes to maintaining Lys1 levels and encourages lysine synthesis.

We were intrigued by one newly identified peroxisomal protein, Ynl097c-b/Pls1, a small open reading frame (smORF) protein. While in the past, smORFs were often overlooked and left uncharacterized, recently increased efforts revealed a range of functions attributed to smORFs [54,55]. One recurring theme for smORFs is that they can modulate protein structure or function by interacting with larger proteins [56]. Our data support this role for Pls1 in tuning Lys1 levels and modulating the lysine biosynthetic pathway. Our work shows that under conditions of lysine depletion, Pls1 concentrates in peroxisomes, acts to stabilize Lys1, and enables cells to produce more internal lysine (Figure 7). Indeed it has been suggested that Lys1 has regulatory mechanisms that are not shared with other enzymes of the lysine biosynthesis pathway [57].

How Pls1 affects Lys1 levels, and lysine metabolism, is yet to be revealed. Pls1 seems to be consistently present in the cytosol. However, in lysine-depleted conditions, both Lys1 and Pls1 are enriched in peroxisomes and Lys1 levels increase [57] partly by Pls1 presence (Figure 6C). We assume that this encourages lysine biosynthesis therefore inside peroxisomes. Indeed, it was shown that Lys1 function in the cytosol is less efficient [50]. We hypothesize that one way for Pls1 to regulate Lys1 abundance is by stabilizing it through the masking of cytosolic degradation signals. Lys1 is predicted to have two potential degrons (regions within a protein responsible for proteasomal degradation), in its loops [58] (Figure S4A). This prediction corresponds to ubiquitination sites detected in Lys1 [59,60]. The masking of such a degron by Pls1 binding would inhibit protein degradation.

While changes in Lys1 levels alone may be enough to explain the effect on saccharopine and lysine levels in Pls1 mutants, an additional mode by which Pls1 might regulate Lys1 (not mutually exclusive with the first) is by affecting its catalytic activity. Lys1 can convert saccharopine to lysine or the reverse reaction depending on the local pH in the active site [52]. Lysine synthesis requires the presence of NAD+ and local pH 10, whereas the reverse reaction requires NADH and local pH 7 [52]. The structure of Lys1 has been determined by X-ray crystallography in its active bound state with NADH and saccharopine [61]. Attempts to model the structure of Pls1 suggest that, on its own, it is likely unstructured [62,63]. The sequence of Pls1 is highly positively charged, including five lysine residues and only one negatively charged glutamate residue. Using ANCHORSmap, a tool that predicts areas of a protein that are prone to strong protein-peptide interaction (low ΔG) named anchoring spots [64], we detected two very low ΔG lysine anchoring spots. These were in the vicinity of the active site, located next to the two glutamate residues implicated in reactant binding and modulation of the pH of the active site [65]. Such binding of lysine residues could partially neutralize the glutamate residues negative charge, modulate the active site-local pH, and substrate binding to reduce lysine degradation (Figure S4B).

More broadly, why would *S. cerevisiae* specifically need a gene that modulates lysine synthesis? Since lysine is one of only three amino acids that *S. cerevisiae* cannot utilize as a nitrogen source [66], it is surprising that it goes to great lengths to store large amounts of lysine [67]. This has been suggested to occur as lysine is utilized for alternative metabolic roles and in response to oxidative stress [67,68,69,70,71]. In addition, evolutionary genetic evidence and the existence of complex regulatory systems demonstrate the importance of lysine in *S. cerevisiae* metabolism [72,73,74,75,76,77]. As peroxisomes contribute to the adjustment of lysine biosynthesis [50], the existence of an element that modulates Lys1 under specific conditions could be part of a regulatory loop disfavoring lysine production in the presence of external lysine while enabling a rapid transition to production during times of need. 

In summary, our work identifies a protein involved in the regulation of lysine synthesis in peroxisomes. Moreover, it demonstrates how this intriguing organelle can underlie central regulatory decisions under changing metabolic requirements in eukaryotic cells and highlights why discovery of the full peroxi-ome is of importance when attempting to understand cellular metabolism. 

## Data Availability

Not applicable.

## Supplementary Materials

The following supporting information can be downloaded at: https://www.mdpi.com/article/10.3390/cells11091426/s1, Figure S1: Sequence conservation of the *YNL097C-B* locus; Figure S2: Overexpression of Lys1 increases localization of Ynl097c-b to peroxisomes without affecting its abundance; Figure S3: Ynl097c-b affects Lys1 levels in lysine deprived medium; Figure S4: Hypotheses as to how Pls1 affects Lys1 stability or function; Table S1: List of strains included in the SWAT *TEF2*pr-mCherry puncta library; Table S2: List of proteins that colocalized with peroxisomes in each screened condition and their targeting factor dependence; Table S3: *S. cerevisiae* strains used in this study; Table S4: Plasmids used in this study [76,77]; Table S5: Primers used in this study; Table S6: Metabolomics data acquired from strains with over expression or deletion of *PLS1* compared to a control strain.
