# Supplementary material for: Pls1 Is a Peroxisomal Matrix Protein with a Role in Regulating Lysine Biosynthesis
Source: Cells. 2022 Apr 22;11(9):1426. doi: 10.3390/cells11091426 (PMC9104712; doi:10.3390/cells11091426)
Supplement: Supplementary file 1 [file cells-11-01426-s001.zip › DavidEtAl_Supplementary_materials.pdf]

David et al. Supplementary Figures

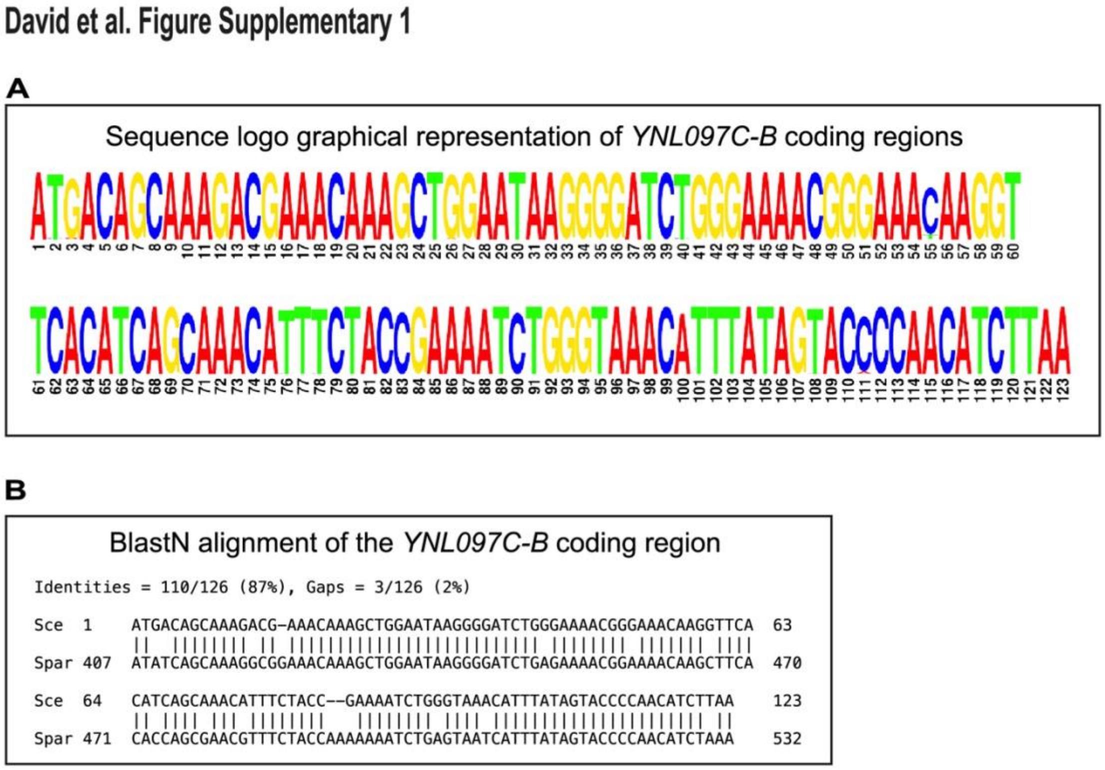

Figure S1: Sequence conservation of the *YNL097C-B* locus

(A) Graphical representation of the sequence logo of multiple alignments made using 857 *YNL097C-B* coding regions from *S. cerevisiae* strains and hybrid species. The alignment demonstrates that the entire coding region of *YNL097C-B* is highly conserved. Not shown are cases of two single base insertions, each in a single locus, between positions 42/43 and 114/115.

(B) BlastN alignment between the *YNL097C-B* coding region of *S. cerevisiae* S288C (*Sce*) and the corresponding region of *S. paradoxus CBS432 (Spar)*. The *Spar* region does not have a 5' ATG codon, a 3' stop codon, and is frame-shifted relative to *Sce* along a central region of 68 bases due to two insertion points. *Spar* coordinates correspond to the position downstream of the Pho23 gene. The sequences used are from Genbank entries BK006947.3:440919-440797 (*Sce*) and CP020255.1:439235-439110 (*Spar*).

David et al. Figure Supplementary 2

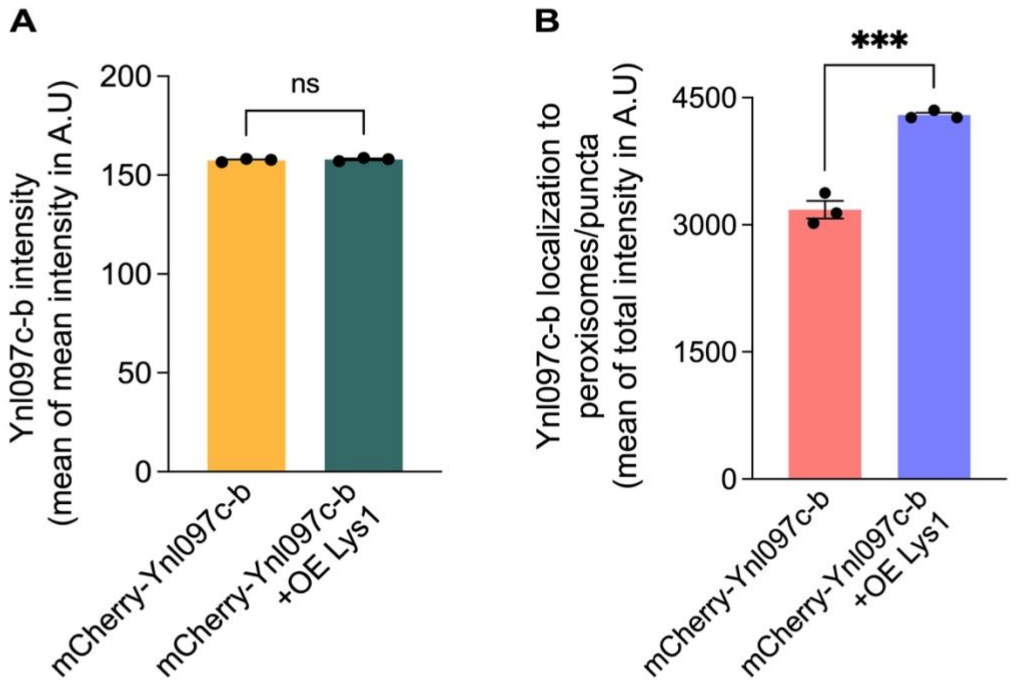

Figure S2: Overexpression of Lys1 increases localization of Ynl097c-b to peroxisomes without affecting its abundance

(A) Quantification of strains used in Fig. 5F for measuring the effect of Lys1 overexpression on the signal intensity (proxy of abundance) of Ynl097c-b. Data are presented as the mean of measured cellular mean intensity (i.e., whole cellular fluorescence divided by cell area), calculated for each of 3 experimental repeats each consisting of hundreds of individual cells. No significant difference in overall signal intensity was detected between strains suggesting that Lys1 expression does not affect the levels of Ynl097c-b. Bars represent the mean with standard error for the three biological repeats.

(B) Quantification of strains used in Fig. 5F for measuring the effect of Lys1 overexpression on the localization of Ynl097c-b to peroxisomes. Data are presented as the mean of total intensity measured for detected mCherry signals inside punctate structures (sub objects), calculated for each of three experimental repeats consisting each of hundreds of individual cells. The significant increase in the total intensity of measured mCherry-Ynl097c-b signals inside punctate structures when Lys1 is overexpressed suggests an increased localization to peroxisomes. Bars represent the mean with standard error for the three biological repeats, *** $p<0.005$.

David et al. Figure Supplementary 3

A

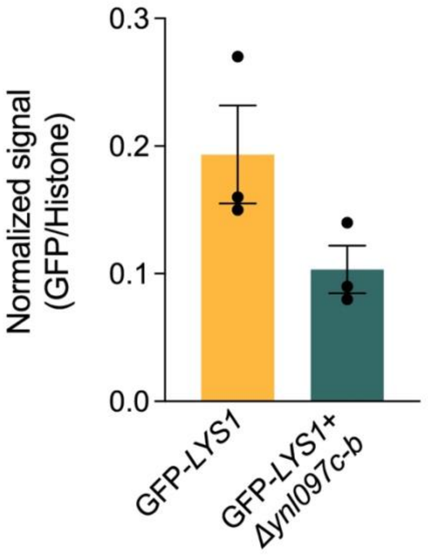

Figure S3: Ynl097c-b affects Lys1 levels in lysine deprived medium. Quantification of the western blot shown in Fig. 6C to demonstrate the effect of Ynl097c-b expression on GFP-Lys1 levels. Data are presented as the detected signal (anti-GFP) normalized to loading control (anti-histone). n=3

David et al. Figure Supplementary 4

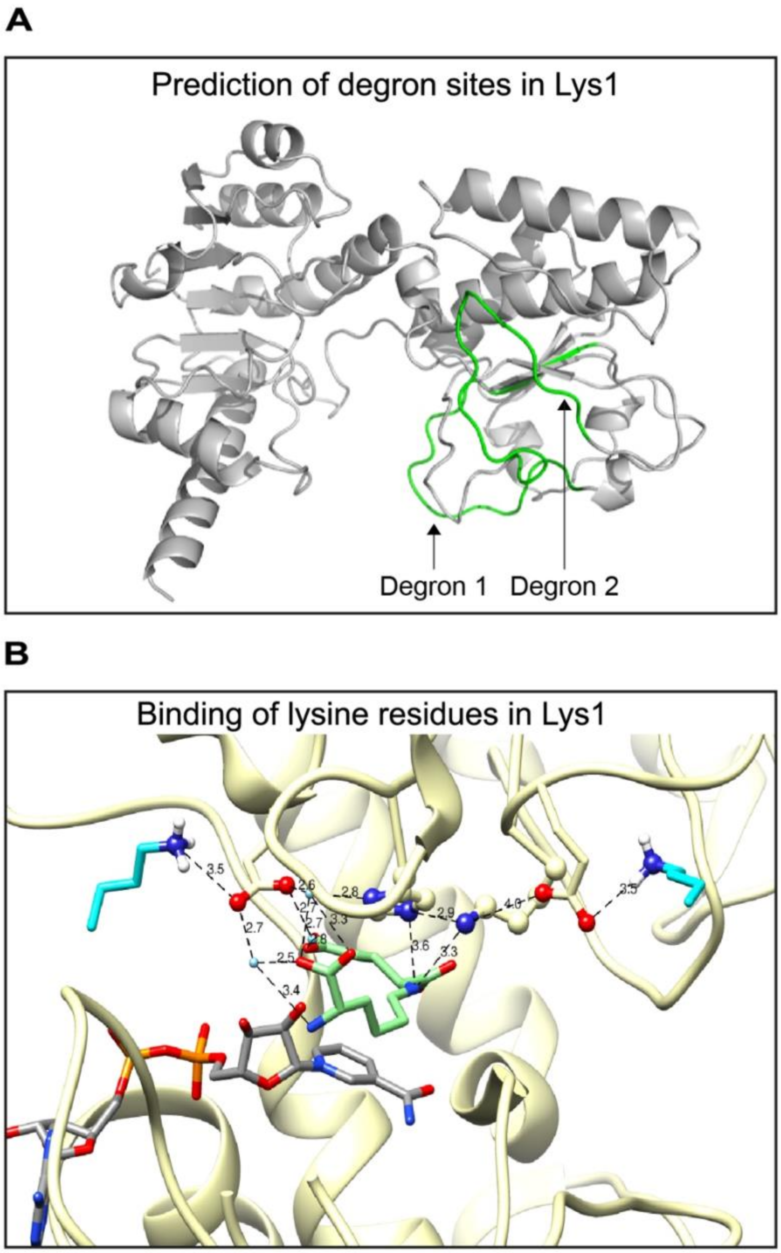

Figure S4: Hypotheses as to how Pls1 affects Lys1 stability or function

(A) The Structure of Lys1 highlighting two potential degron sites (as predicted [58]) which may serve to destabilize it in the cytosol.

(B) Binding of lysine residues to Lys1. The location of two low ΔG lysine anchoring spots near E122 and E78 of Lys1 (-12.7 and -7.4 kcal/mol, respectively), as detected with ANCHORSmap. The binding of positively charged residues in these locations likely affects the local pH of the active site and the binding of reactants, and thus may modulate the directionality of the reversible lysine synthesis/degradation reaction. Shown is the active site of Lys1 with bound NADH and saccharopine (PDB entry 3UH1). The active

residues K77 and H96 are shown in ball-and-stick with pale-yellow carbon atoms, E122, E78, NADH and saccharopine are shown in stick diagram with pale yellow, gray and green carbon atoms, respectively. Oxygen, nitrogen and hydrogen atoms are shown in red, blue and white, respectively. The two lysine anchors are shown in cyan with the terminal NH_3 groups emphasized in ball and stick. Three water molecules that mediate the interaction of E122 and saccharopine are shown as small light blue balls. Hydrogen bonds, including the long E78-K77 hydrogen bond, are indicated; distances are given in Å.
